# Combating the SARS-CoV-2 Omicron (BA.1) and BA.2 with potent bispecific antibodies engineered from non-Omicron neutralizing antibodies

**DOI:** 10.1038/s41421-022-00463-6

**Published:** 2022-10-07

**Authors:** Yingdan Wang, Xiang Zhang, Yunping Ma, Yanqun Wang, Wuqiang Zhan, Qinwen Zheng, Meng Zhang, Ping Ji, Mei Liu, Qianying Liu, Tingting Sun, Tongyu Zhu, Yumei Wen, Lei Sun, Jincun Zhao, Fan Wu, Zhenguo Chen, Jinghe Huang

**Affiliations:** 1 Key Laboratory of Medical Molecular Virology (MOE/NHC/CAMS) and Shanghai Institute of Infectious Disease and Biosecurity, the Fifth People’s Hospital of Shanghai, Shanghai Public Health Clinical Center, Institutes of Biomedical Sciences, School of Basic Medical Sciences, Fudan University, Shanghai, China; 2 Shanghai Immune Therapy Institute, Shanghai Jiao Tong University School of Medicine Affiliated Renji Hospital, Shanghai, China; 3 State Key Laboratory of Respiratory Disease, National Clinical Research Center for Respiratory Disease, Guangzhou Institute of Respiratory Health, the First Affiliated Hospital of Guangzhou Medical University, Guangzhou, Guangdong China; 4 Institute of Infectious Disease, Guangzhou Eighth People’s Hospital of Guangzhou Medical University, Guangzhou, Guangdong China; 5 Guangzhou Laboratory, Bio-island, Guangzhou, Guangdong China

**Keywords:** Cryoelectron microscopy, Mechanisms of disease

## Abstract

The highly mutated and transmissible Omicron (BA.1) and its more contagious lineage BA.2 have provoked serious concerns over their decreased sensitivity to the current COVID-19 vaccines and evasion from most anti-SARS-CoV-2 neutralizing antibodies (NAbs). In this study, we explored the possibility of combating the Omicron and BA.2 by constructing bispecific antibodies based on non-Omicron NAbs. We engineered 10 IgG-like bispecific antibodies with non-Omicron NAbs named GW01, 16L9, 4L12, and REGN10987 by fusing the single-chain variable fragments (scFvs) of two antibodies through a linker and then connecting them to the Fc region of IgG1. Surprisingly, 8 out of 10 bispecific antibodies showed high binding affinities to the Omicron receptor-binding domain (RBD) and exhibited extreme breadth and potency against pseudotyped SARS-CoV-2 variants of concern (VOCs) including Omicron and BA.2, with geometric mean of 50% inhibitory concentration (GM IC_50_) values ranging from 4.5 ng/mL to 103.94 ng/mL, as well as the authentic BA.1.1. Six bispecific antibodies containing the cross-NAb GW01 not only neutralized Omicron and BA.2, but also neutralized the sarbecoviruses including SARS-CoV and SARS-related coronaviruses (SARSr-CoVs) RS3367 and WIV1, with GM IC_50_ ranging from 11.6 ng/mL to 103.9 ng/mL. Mapping analyses of 42 spike (S) variant single mutants of Omicron and BA.2 elucidated that these bispecific antibodies accommodated the S371L/F mutations, which were resistant to most of the non-Omicron NAbs. A cryo-electron microscopy (cryo-EM) structure study of the representative bispecific antibody GW01-16L9 (FD01) in its native full-length IgG form in complex with the Omicron S trimer revealed 5 distinct trimers and one novel trimer dimer conformation. 16L9 scFv binds the receptor-binding motif (RBM), while GW01 scFv binds a epitope outside the RBM. Two scFvs of the bispecific antibody synergistically induced the RBD-down conformation into 3 RBD-up conformation, improved the affinity between IgG and the Omicron RBD, induced the formation of trimer dimer, and inhibited RBD binding to ACE2. The trimer dimer conformation might induce the aggregation of virions and contribute to the neutralization ability of FD01. These novel bispecific antibodies are strong candidates for the treatment and prevention of infection with the Omicron, BA.2, VOCs, and other sarbecoviruses. Engineering bispecific antibodies based on non-Omicron NAbs could turn the majority of NAbs into a powerful arsenal to aid the battle against the pandemic.

## Introduction

Omicron (BA.1), first identified in South Africa and reported to the WHO at the end of Nov. 2021^1^, became the dominant severe acute respiratory syndrome coronavirus 2 (SARS-CoV-2) variant globally in Jan. 2022. Omicron variant has mutated into four sublineages: Omicron (BA.1), BA.1.1, BA.2, and BA.3. BA.1 has 34 mutations in the spike protein. BA.1.1, BA.2, and BA.3. have 35, 29, and 30 spike mutations, respectively (Supplementary Table S1). BA.2 is more contagious than Omicron and became more prevalent than Omicron since Jan. 2022 in multiple countries^2^. BA.3 prevalence has remained low compared to Omicron and BA.2. Omicron variant and its sublineages extensively escape neutralization by sera from vaccinated or convalescent individuals^3–11^. Moreover, most neutralizing antibodies (NAbs)^12–18^, including many clinical-stage monoclonal antibodies (mAbs)^19–25^, have completely lost their neutralization potency against Omicron^3,5,6,26^. Thus far, only one clinical-stage mAb, bebtelovimab, was reported to be able to neutralize all four sublineages^5^. Therefore, there is an urgent need to explore and develop countermeasures against the Omicron variants.

In this study, we used four NAbs named REGN10987^19^, GW01^27^, 16L9, and 4L12, which failed to bind or neutralize the Omicron, to engineer full-length IgG-like bispecific antibodies. We surprisingly found that these bispecific antibodies could neutralize all the variants of concern (VOCs), including Omicron, BA.2, and authentic BA.1.1, while the parental antibody cocktail showed no neutralization against Omicron. Cryo-electron microscopy (cryo-EM) structure study showed six dynamic states of the Omicron spike (S) trimer upon bispecific antibody binding, including a novel trimer dimer conformation, within which RBDs were all in “up” conformations. This trimer dimer is critical for inhibiting ACE2 binding and explains the superiority of the bispecific antibody. These novel bispecific antibodies are strong candidates for the treatment and prevention of infection with the VOCs and other sarbecoviruses that may cause future emerging or reemerging coronavirus diseases.

## Results

### Isolation of three non-Omicron NAbs from COVID-19 convalescent individuals

**Fig. 1:**
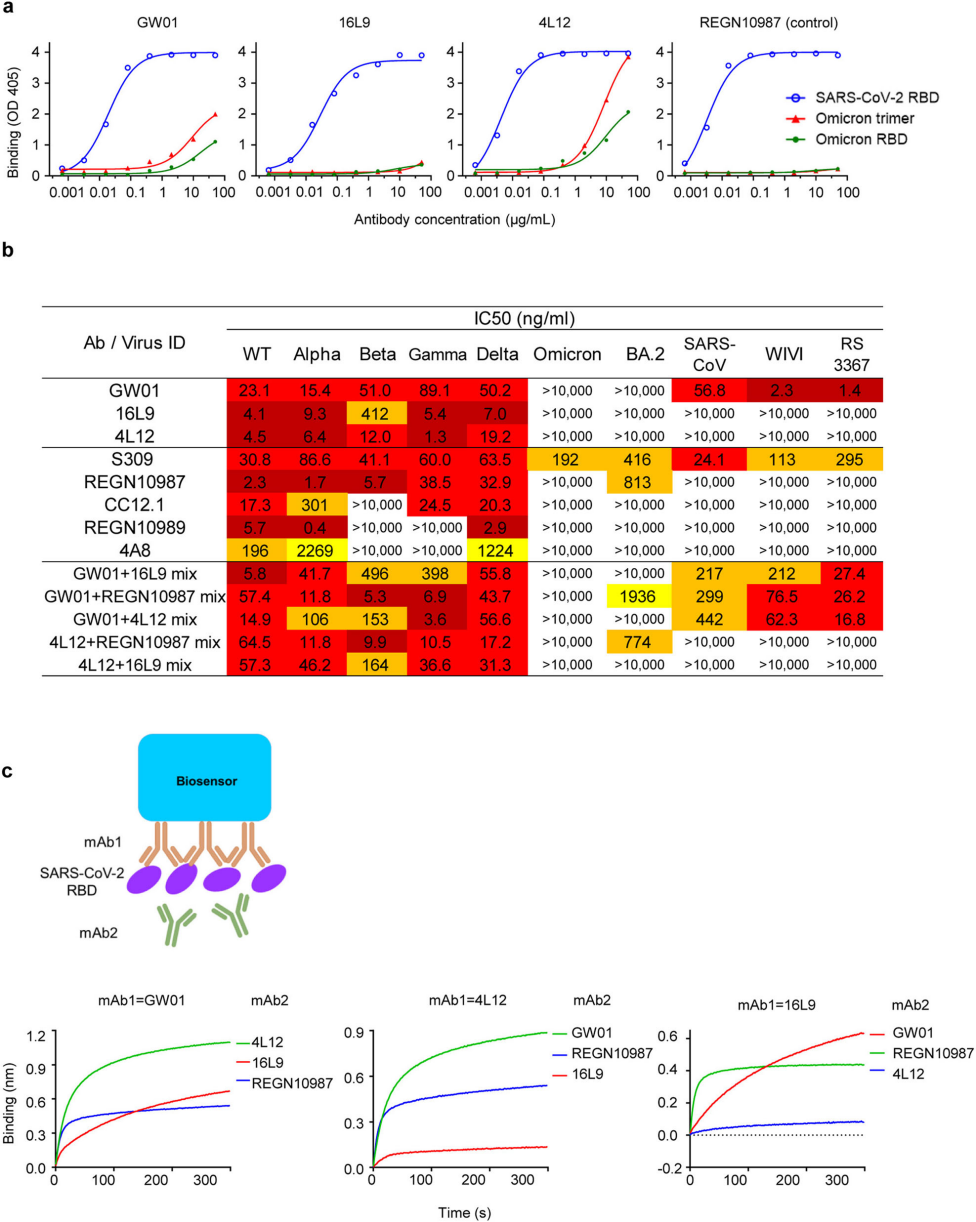
Isolation of three non-Omicron NAbs from COVID-19 convalescent individuals. **a** Binding of GW01, 16L9, and 4L12 to the SARS-CoV-2 RBD, Omicron RBD and trimer in an ELISA. REGN10987 was used as a control. **b** Neutralizing activities of GW01, 16L9, and 4L12 and their combinations against pseudotyped SARS-CoV-2 and its variants Alpha, Beta, Gamma, Delta, and Omicron, as well as sarbecoviruses. S309 was used as a positive control. **c** Binding of 4L12, 16L9, and RGN10987 to the SARS-CoV-2 RBD in competition with GW01, as measured by BLI.

We sorted and cultured SARS-CoV-2 S-specific memory B cells from two recovered COVID-19 patients and discovered three anti-SARS-CoV-2 NAbs, designated GW01, 4L12, and 16L9. The germlines and CDR3s of these antibodies are listed in Supplementary Table S2. All three antibodies showed strong binding to the RBD of SARS-CoV-2 (Fig. 1a). However, they had no or weak binding to the S trimer or S RBD of the Omicron variant (Fig. 1a).

GW01, 4L12, and 16L9 potently neutralized SARS-CoV-2 and the VOCs Alpha, Beta, Gamma, and Delta, but they failed to neutralize the Omicron and BA.2 (Fig. 1b). A panel of control NAbs failed to neutralize the Omicron and BA.2 except S309. S309 neutralized Omicron and BA.2 to a similar degree as previous reports^28,29^. REGN10987 weakly neutralized BA.2 at an IC_50_ of 813 ng/mL. GW01 was a cross-NAb that was able to neutralize SARS-CoV and the SARS-related coronaviruses (SARSr-CoVs) RS3367 and WIV1. GW01 showed no competition with 4L12, 16L9, or the control antibody REGN10987 in binding the RBD (Fig. 1c), indicating that GW01 binds to an epitope different from that bound by 4L12, 16L9, and REGN10987. However, combinations of two antibodies targeting different epitopes showed no increasement of neutralization against the Omicron and BA.2 (Fig. 1b).

### Binding and neutralization of the Omicron variant by bispecific antibodies

**Fig. 2:**
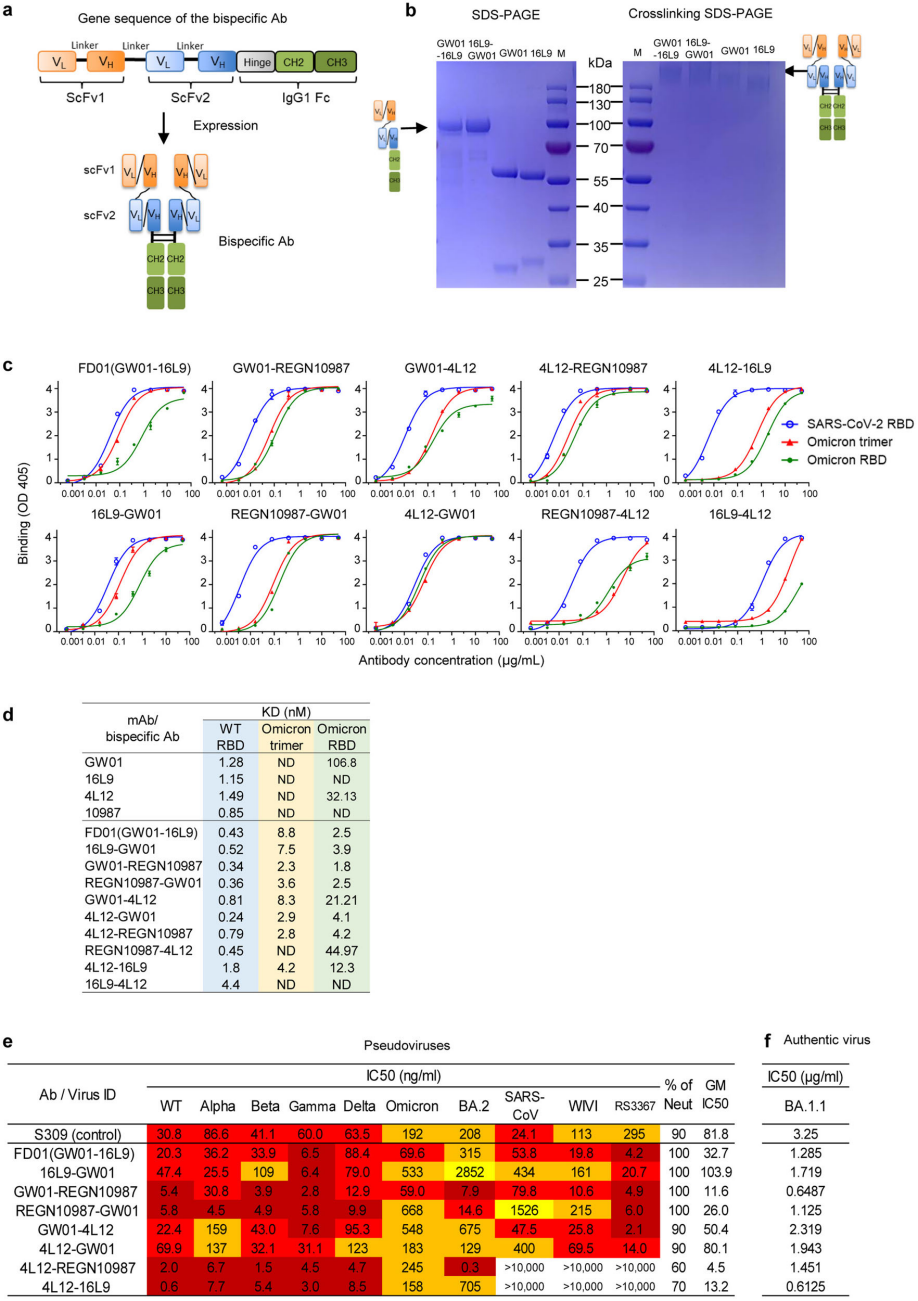
Binding and neutralization of the Omicron variant by bispecific antibodies. **a** Schematic diagrams showing the structures of bispecific antibodies. **b** SDS-PAGE and cross-linking SDS-PAGE gels showing the sizes of the representative bispecific antibodies and their parental antibodies. **c** Binding specificities of the bispecific antibodies to the SARS-CoV-2 RBD-His, Omicron trimer-His, or RBD-His protein. **d** Binding affinities of the bispecific antibodies to the SARS-CoV-2 RBD-His, Omicron trimer-His, or RBD-His protein were measured by BLI experiments. ND represents not determined. **e** Neutralization by bispecific antibodies against the SARS-CoV-2 VOCs, including Omicron, BA.2, and sarbecoviruses. **f** Neutralization of bispecific antibodies against authentic BA.1.1 virus.

We constructed bispecific antibodies targeting different epitopes in the RBD using GW01 in combination with 16L9, 4L12, and REGN10987, and explored their possibilities to neutralize Omicron variants. We linked the single-chain variable fragments (scFvs) of the parental antibodies with a GlySer(Gly_4_Ser)_4_ linker and then fused them to a hinge-CH2-CH3 fragment of human immunoglobulin (hIgG1 Fc) to generate a single gene-encoded IgG-like bispecific antibody (Fig. 2a). For example, the sequence order of the GW01-16L9 (FD01) bispecific antibody was as follows: GW01 VL-(Gly_4_Ser)_3_-GW01 VH-GlySer(Gly_4_Ser)_4_-16L9 VL-(Gly_4_Ser)_3_-16L9 VH-hinge-CH2-CH3. SDS-PAGE results showed that the size of the single chain of two representative bispecific antibodies, GW01-16L9 (FD01) and 16L9-GW01, was ~100 kDa and that the purity was > 95% (Fig. 2b). Cross-linking bispecific antibodies with glutaraldehyde revealed that the full size of the bispecific antibodies was ~200 kDa, which was 10% larger than that of the parental antibodies (180 kDa, Fig. 2b).

We constructed ten bispecific antibodies and tested their binding abilities to the RBD or S trimer of SARS-CoV-2 and the Omicron. We demonstrated that eight bispecific antibodies, FD01 (GW01-16L9), 16L9-GW01, GW01-REGN10987, REGN10987-GW01, GW01-4L12, 4L12-GW01, 4L12-REGN10987, and 4L12-16L9, strongly bound to the RBDs of SARS-CoV-2 and Omicron (Fig. 2c), and had high binding affinities to these proteins (Fig. 2d; Supplementary Fig. S1). Consistent with the ELISA results, GW01 and 4L12 showed low binding affinities to the Omicron RBD, while 16L9 and REGN10987 showed no binding. These results indicated that the joint structure of the bispecific antibody increases the parental antibody binding affinity to the Omicron RBD.

To understand the breadth of these bispecific antibodies, we performed a neutralization assay using SARS-CoV-2 pseudoviruses, including Alpha, Beta, Gamma, Delta, Omicron, and BA.2, and the sarbecoviruses SARS-CoV, WIV1, and RS3367. Surprisingly, these eight bispecific antibodies potently neutralized the Omicron and BA.2. Six bispecific antibodies containing the cross-NAb GW01 strongly neutralized all the tested VOCs and sarbecoviruses (Fig. 2e). FD01 and GW01-REGN10987 were the most broad NAbs, with geometric mean (GM) IC_50_ values of 32.7 ng/mL and 11.6 ng/mL, respectively. 4L12-REGN10987 neutralized all the VOCs with a GM IC_50_ value of 4.5 ng/mL. It potently neutralized BA.2 at the concentration of 0.3 ng/mL. Taken together, these data indicated that bispecific antibodies consisting of non-Omicron NAbs efficiently neutralize Omicron and BA.2 in a way that is different from the antibody cocktail.

To confirm the neutralization efficacy of the bispecific antibodies, we performed plaque reduction neutralization assays with an authentic Omicron variant containing the R346K mutation (BA.1.1), which escapes more SARS-CoV-2 NAbs than the Omicron^5^. All eight bispecific antibodies efficiently neutralized the authentic BA.1.1 virus (Fig. 2f) and exhibited better potency than S309 (IC_50_ = 3.25 μg/mL). These data confirmed that the bispecific antibodies composed of non-Omicron NAbs are able to neutralize the Omicron.

### Bispecific antibodies accommodated the S371L/F escape mutations in the Omicron and BA.2

We constructed 42 single mutants of the Omicron and BA.2 (eight single mutants unique to BA.2 were highlighted in blue) to identify the key residues that mediate resistance to GW01, 16L9, 4L12, and REGN10987. The K417N mutation resulted in complete resistance to 16L9 (> 1000-fold, Table 1), which explained the reduction of neutralizing activity of 16L9 against Beta variant. The S375F, N440K, and G446S mutations could completely abolish the neutralizing capacity of REGN10987 (> 1000-fold). The S371L mutation in Omicron and S371F mutation in BA.2, which were found to stabilize the RBD-down conformation, greatly decreased the neutralization activities of the majority of NAbs^28,30^. S371L mutation greatly decreased the neutralization activities of GW01, 4L12, and REGN10987 (74.1- to > 493-fold), while S371F mutation was sensitive to GW01, 16L9, and 4L12, and only decreased the neutralization activities of REGN10987 (74.1-fold, Table 1). However, all five tested bispecific antibodies showed a slight decrease (12.4–15.6-fold) or no change in neutralization activity against the S371L/F mutations. Therefore, bispecific antibodies accommodate the S371L/F escape mutations of the Omicron and BA.2, resulting in extraordinary breadth and potency.

**Table 1:**
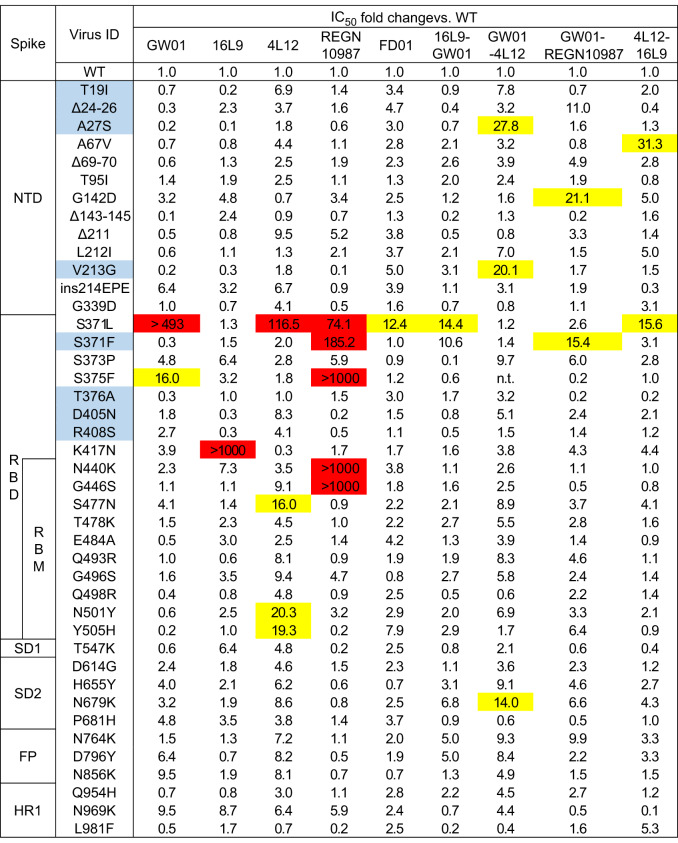
Neutralization by GW01, 16L9, 4L12, REGN10987, and five bispecific antibodies against 42 variants containing single mutation present in the Omicron and BA.2.

Mutations unique to BA.2 are highlighted in light blue. Fold change is defined as the IC_50_ of the mutant/the IC_50_ of WT. Mutations that resulted in fold change values between 10 and 50 are highlighted in yellow, and those with values > 50 are highlighted in red.

### Cryo-EM structures of Omicron S trimer and bispecific antibody FD01 (GW01-16L9) IgG

**Fig. 3:**
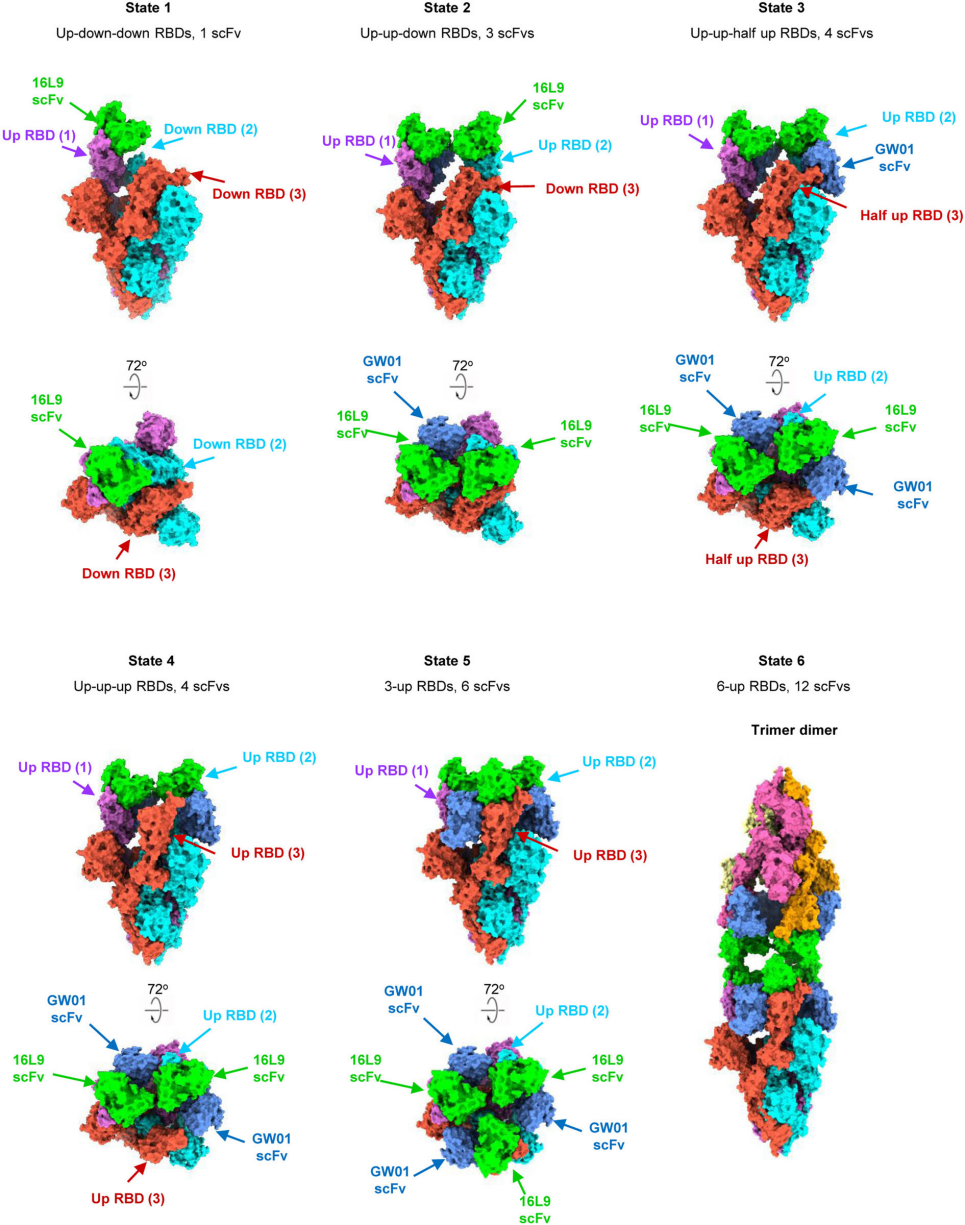
Cryo-EM structures of the Omicron S trimer in complex with the bispecific antibody FD01 IgG. Cryo-EM structure of the prefusion stabilized SARS-CoV-2 Omicron S ectodomain trimer in complex with the bispecific antibody GW01-16L9 (FD01) IgG, revealing 6 states of the complex. State 1: up-down-down RBDs, 1 scFv, 3.47 Å. State 2: up-up-down RBDs, 3 scFvs, 3.70 Å. State 3: up-up-half-up RBDs, 4 scFvs, 3.91 Å. State 4: up-up-up RBDs, 4 scFvs, 3.47 Å. State 5: 3-up RBDs, 6 scFvs, 3.87 Å. State 6: trimer dimer, 6-up RBDs, 12 scFvs, 6.11 Å. Two perpendicular views of Omicron S–FD01 are shown in surface representation, with 16L9 ScFv in lime and GW01 ScFv in cornflower blue.

To further investigate the neutralization mechanism of the bispecific antibodies, we chose FD01 (GW01-16L9) as a representative antibody for the structural study. We determined the cryo-EM structure of the prefusion stabilized SARS-CoV-2 Omicron S ectodomain trimer in complex with the bispecific antibody GW01-16L9 (FD01) in its native full-length IgG form, revealing 6 states of the complex with resolutions ranging from 3.47 Å to 6.11 Å (Fig. 3; Supplementary Figs. S2, S3). Local-refinement focusing on the RBD and scFvs improved the interface region to 3.51 Å resolution and allowed us to unambiguously build the RBD and scFvs (Supplementary Fig. S4, Table S3).

**Fig. 4:**
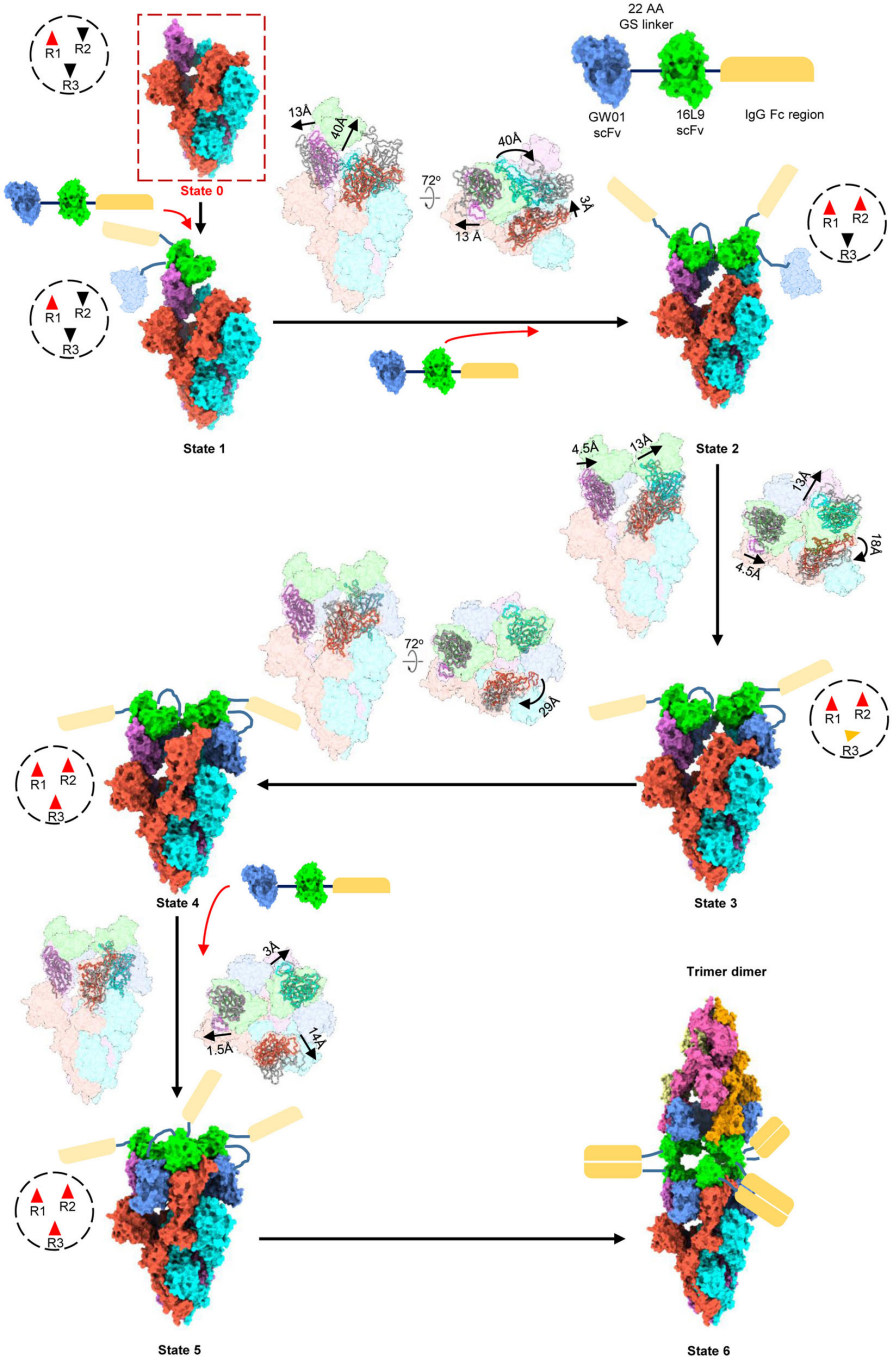
Conformation transitions of Omicron S–FD01 in all states. The representative bispecific antibody GW01-16L9 (FD01) IgG in complex with the Omicron S trimer. One 16L9 binds to the “up” RBD-1 in the apo S trimer (State 0) to form the State 1 complex. Then, the 22-aa GS linker between 16L9 and GW01 guides GW01 to bind to RBD-1 and push it more open to accommodate GW01, which unlocks the “down” RBD-2 and induces it to the “up” state (State 2). Thus, another 16L9 could easily catch the “up” state RBD-2. The same triggering process then occurs on RBD-2 (State 3 and State 4) and RBD-3 (State 5), allowing the binding of the second and third FD01 to RBD-2 and RBD-3, respectively. Thus, 16L9 scFv and GW01 scFv synergistically induced the RBD-down conformation into 3 RBD-up conformation. Finally, three pairs of Fc regions from FD01s cross-link two trimers and form a trimer dimer (State 6). Black dotted circles show the states of three RBDs in a spike trimer: red triangles represent "up" state, black triangles represent "down" state and the orange one represents "half-up" state. The direction and distance of movement of RBDs from one state to next state is marked.

These six cryo-EM structures represent the conformational transitions of the Omicron S trimer upon FD01 binding. To simplify the presentation, three protomers of a S trimer are clockwisely defined as 1, 2, and 3 (Figs. 3, 4; Supplementary Video S1). The apo S trimer (state 0) includes one regular up RBD and two down RBDs. In state 1, only one 16L9 binds to the “up” RBD (RBD-1) of the apo S trimers. Following that, GW01 binds to the “up” RBD-1, inducing it to a wide-up state (via an ~13 Å outward motion) and pushing the “down” RBD-2 to the “up” state to bind the second 16L9 (state 2). Then, in state 3, the “up” RBD-2 further moves outward to the “wide-up” state, allowing the second FD01 binding and pushing the “down” RBD-3 to a “half-up” state. In state 4, the “half-up” RBD-3 opens up to the regular “up” state, ready for 16L9 binding. Then, in state 5, the third FD01 binds to RBD-3 and induces RBD-3 to the “wide-up” state. Finally, three FD01s cross-link two trimers in state 5, forming a trimer dimer conformation (state 6). Thus, the six states represent the continuous conformational transitions. In addition to the motion of RBDs, the N-terminal domains (NTDs) of the trimer also move slightly following the motion of their neighboring RBDs.

Negative stain images of Omicron S trimer–FD01 (scFv)2 and Omicron S trimer–FD01 IgG showed that only full-length IgG FD01 induced the formation of trimer dimer (Supplementary Fig. S5), indicating that the Fc region is essential for the formation of trimer dimer and the neutralizing activity of FD01.

### FD01 targets one epitope in RBM and one epitope outside of RBM

**Fig. 5:**
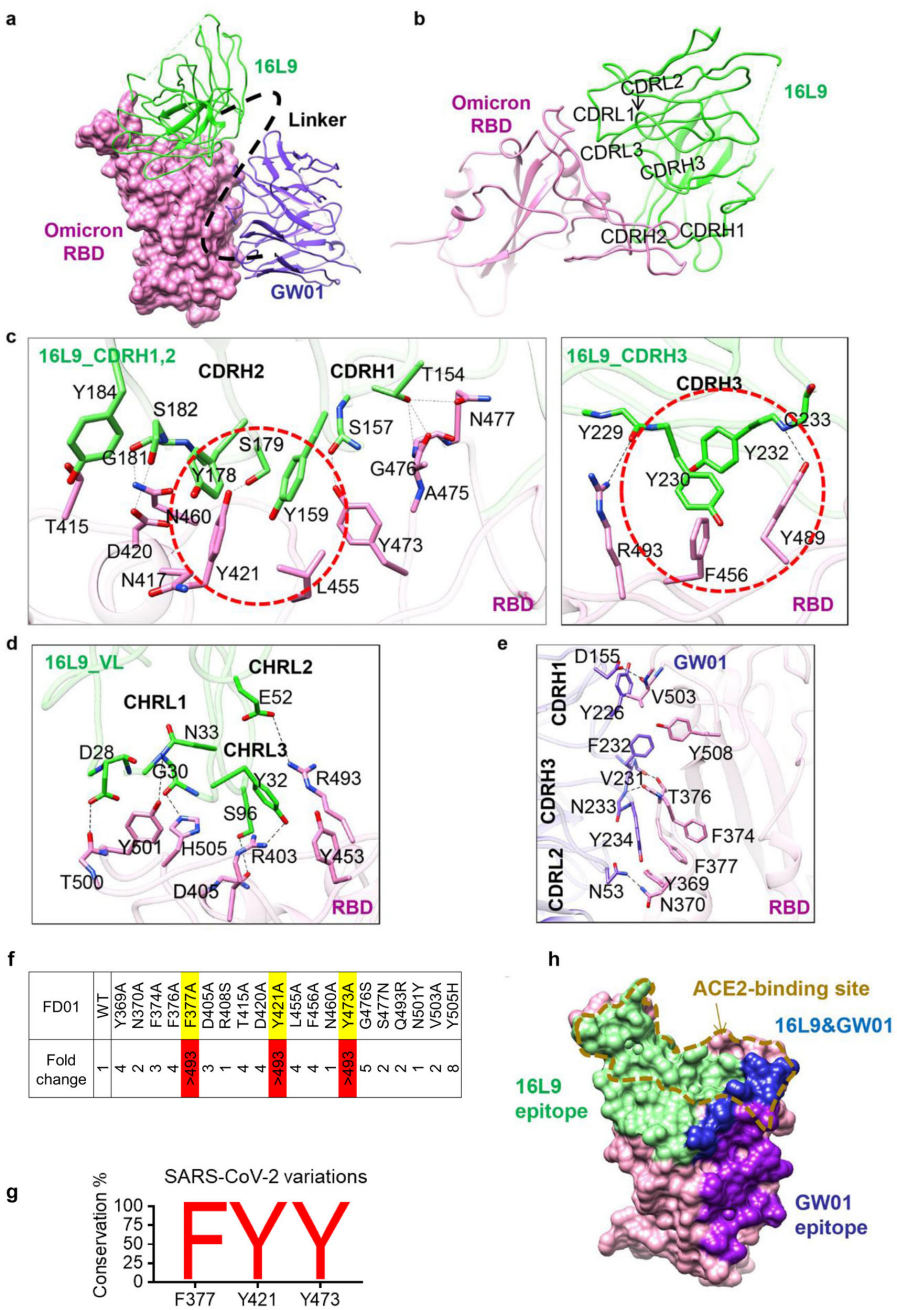
Two conserved epitopes recognized by FD01. **a** Close-up view of the interaction between FD01 and Omicron. The Omicron S RBD is displayed in pink surface. 16L9 and GW01 are shown as cartoons colored green and medium-blue, respectively. **b** The interactions between S RBD and 16L9. **c** The detailed interaction between RBD and the heavy chain of 16L9. The residues involved in interactions are represented as sticks. The polar interactions are indicated as dotted lines. The hydrophobic interactions are highlighted by red circles. **d** The detailed interaction between RBD and the light chain of 16L9. **e** The detailed interaction between RBD and GW01. **f** Epitope mapping of FD01. **g** Conservancy analysis of FD01 epitope. **h** Surface representation of RBD showing the buried binding site, including 16L9 (green), GW01 (purple), and 16L9 epitope overlapping with GW01 region (blue); goldenrod dotted line indicates receptor-binding site.

16L9 and GW01 bind to two different sites of one RBD (Fig. 5a). The epitope of 16L9 mostly overlaps with the RBM, while GW01 binds outside of the RBM. The binding of 16L9 with the RBD buries a 1061 Å^2^ surface area, a total of 35 residues from RBD are involved. The interaction between 16L9 and RBD is largely driven by extensive hydrophilic and hydrophobic interactions between CDRH1, CDRH2, CDRH3, and CDRL1 of 16L9 and RBD (Fig. 5b). Residues D405, T415, D420, Y421, Y453, L455, F456, Y473, A475, N477, Y489, R493, T500, Y501, and H505 of the RBD are involved in this interaction, forming 14 pairs of hydrogen bonds and 3 patches of hydrophobic interactions (Fig. 5c). K417 is located at the epitope of 16L9. the K417N mutation might disrupt its interaction with 16L9 because the side chain becomes shorter (Fig. 5c). In addition, the hydrogen bond between S96 of CDRL3 and R403 from the RBD and the salt bridge between E52 of CDRL2 and R493 from the RBD further enhance the interaction (Fig. 5d). Strikingly, the 16L9 binding site shared 19 epitope residues with RBD binding site, including the key residues Y453, A475, Y489, R493, T500, Y501, and H505 required for ACE2 recognition and binding^31^ (Fig.  5h). The neutralization activity test showed that 16L9 alone was able to broadly neutralize SARS-CoV-2 and SARS-CoV-2 variants except K417N mutant, Omicron and BA.2. Thus, the mutations K417N, S477N, Q493R, and Y505H in Omicron, which are located at 16L9 binding site (Supplementary Fig. S6), reduced the binding affinity between Omicron RBD and 16L9 (Fig. 2d), making 16L9 alone fail to neutralize Omicron.

The binding of GW01 to RBD buries a surface area of 668.2 Å. The interaction between GW01 and the RBD is mainly contributed by CDRH3. The long loop (226-YGPPDVFNY-234) of CDRH3 engages with Y369, F374, T376, F377, Y508, and V503 from the RBD, forming 3 patches of hydrophobic interactions and 2 pairs of hydrogen bonds (Fig. 5e). Resides D155 on CDRH1 and N53 on CDRL2 are also involved in the interaction by forming hydrogen bonds between V503 and N370, respectively (Fig. 5e). Omicron mutations S373P, S375F, and Y505H locating in GW01 binding site (Supplementary Fig. S6) may cause Omicron variant to escape the neutralization by GW01 alone (Fig. 1b). S371 is adjacent to the epitope of GW01 (Supplementary Fig. S6). S371L mutation might affect the interaction between RBD and GW01 by changing the conformation of loop 370–375. However, when these mutations were combined together, the affinity between FD01 and Omicron was increased (Fig. 2d) and FD01 was able to broadly neutralize SARS-CoV-2 and its variants (Fig. 2e). Thus, neutralization of these variants suggests that combining 16L9 and GW01 to form FD01 makes the binding interface more resistant to these mutations.

To verify the key epitope of FD01, we analyzed the RBD residues involved in binding FD01 (Supplementary Fig. S6) and constructed 20 RBD single mutants by mutating RBD residues that play important roles in FD01 binding. F377A, Y421A, and Y473A mutations completely abolished FD01 neutralization (Fig. 5f). F377 is a key residue in the hydrophobic patch of RBD recognized by GW01 (Fig. 5e). Residues Y421 and Y473 form hydrogen bonds with 16L9 (Fig. 5c). Thus, residues F377, Y421 and Y473 were very important for FD01 recognition and interaction. These three residues are 99.99% conserved in SARS-CoV-2 (Fig. 5g), indicating that FD01 binds a very conserved epitope.

### Collaborative binding mechanism of the FD01 bispecific antibody

All five representative bispecific antibodies prevented RBD binding to ACE2 protein in the biolayer interferometry (BLI) competition assay, while the control antibody S309 did not affect the S/ACE2 interaction (Supplementary Fig. S7). FD01 showed a strong inhibitory effect against ACE2 binding. Structural alignment of the FD01–RBD complex with the ACE2–RBD complex indicated that both 16L9 and GW01 were able to compete with ACE2 when binding to the RBD (Fig. 5h), which was consistent with the competition assay (Supplementary Fig. S7).

Both 16L9 and GW01 could neutralize all the other VOCs except Omicron, probably by occupying the ACE2-binding site. Mutations in the Omicron S RBD affect the neutralization activity of 16L9 and GW01 when these antibodies are used alone. However, the bispecific antibody FD01 (GW01-16L9) can work as a functional NAb targeting Omicron. Our structures hint this collaborative binding mechanism.

First, one 16L9 binds to the exposed epitope of “up” RBD-1 in the apo Omicron S trimer as a trigger. Then, the 22-aa GS linker between 16L9 and GW01 guides GW01 to bind to RBD-1 and push it more open, which unlocks the “down” RBD-2 and induces it to the “up” state (Fig. 4). Thus, another 16L9 could easily catch the “up” state RBD-2. The same triggering process then occurs on RBD-2 and RBD-3, allowing the binding of the second and the third FD01 to RBD-2 and RBD-3, respectively.

Thus, although either 16L9 or GW01 alone could not bind Omicron S tightly, the GW01-16L9 bispecific antibody could bind Omicron S collaboratively with the help of each other (Supplementary Fig. S8). The neutralization mechanism of FD01 may be mediated by the unique engineering of the combination of two antibodies into one, which enlarges the interface area, improves the affinity of a single IgG and the RBD, accommodates the S371L mutation, stabilizes the interaction by additional contacts between the two antibodies, and therefore blocks the infection of SARS-CoV-2 Omicron and BA.2.

## Discussion

The recently emerged SARS-CoV-2 Omicron variants raised unprecedented global concern about the invalidation of most FDA-approved antibody drugs individually or in combination, including LY-CoV555, LY-CoV016, REGN10933, REGN10987, AZD8895, and AZD1061^31^. It has been reported that tandem bi-bNAb PGT28-iMAb (BiIA-SG) targeting different epitopes of HIV-1 increased the neutralizing breadth and potency than their parental antibodies for their synergistic effects^32^. Using the NAbs that failed to neutralize the Omicron variant, we constructed a series of novel bispecific antibodies that are capable of neutralizing all SARA-CoV-2 VOCs, including the Omicron and BA.2. Interestingly, single IgG parental antibodies or the combination of parental antibodies failed to neutralize Omicron, although the neutralization activity against SARS-CoV-2 or SARS-CoV-2 Alpha, Beta, Gamma, and Delta was remarkable. Thus, the effective neutralization of Omicron variants by bispecific antibodies may be due to the construction of this bispecific antibody.

This study revealed the cryo-EM structure of the bispecific antibody FD01 in its native full-length IgG form in complex with Omicron S trimer. Although numerous studies have documented high-resolution structures of the Fab and S trimer complexes, the IgG–S trimer complex structure is rare because of the sample preference for aggregation and the flexibility of Fc. The high-resolution structures of the bispecific antibody IgG in complex with Omicron S trimer are undoubtedly important for understanding of its neutralizing mechanisms against Omicron and BA.2.

The ability of antibodies to induce the RBD in the “up” conformation was proved to be important for neutralizing the Omicron variant. Zhou et al. proved that the antibody combination with improved Omicron neutralization (B1-182.1/A19-46.1) synergistically induced the 3-RBD-up conformation^28^. A broad NAb 6M6 neutralized the Omicron infection through a novel trimer dimer conformation by interacting with 6 RBDs and inducing 6-up RBDs^33^. The cryo-EM structures of Omicron S–FD01 unveiled 5 distinct trimers and one unique trimer dimer conformation. From different states of the Omicron S–FD01 complex, we revealed that two scFvs of the bispecific antibody synergistically bind to two distinct epitopes and induce the RBD-down conformation into 3-RBD-up conformation. Fc region of IgG is important for the conformational change. The novel engineering approach of combining two antibodies into one enlarged the interface area and improved the affinity between a single IgG and the RBD, which may account for the neutralizing mechanism mediated by FD01.

Most SARS-CoV-2 bispecific antibodies were constructed with SARS-CoV-2-specific NAbs^34^. However, construction of bispecific NAbs using cross-NAb against sarbecoviruses has not been reported so far. All six bispecific antibodies constructed with cross-NAb GW01 in this study potently neutralized all VOCs as well as other sarbecoviruses, including SARS-CoV and SARSr-CoVs from bats. Therefore, our technique may lead to the development of the broadest-spectrum of pan sarbecovirus NAbs for the forthcoming sarbecoviruses.

Taken together, this study reveals a novel trimer dimer conformation and a novel neutralization mechanism that inhibits the infection of the Omicron and BA.2. Engineering bispecific antibodies with non-Omicron NAbs provide a new way to design antibody drugs against Omicron variants and the upcoming SARS-CoV-2 variants, which may aid the battle against the pandemic.

## Materials and methods

### Cell lines, proteins, viruses, and plasmids

The primary human embryonic kidney cell lines (HEK293T) and Huh-7 cells were cultured in Dulbecco’s modified Eagle’s medium (DMEM; Gibco, Grand Island, NY, USA) with 10% fetal bovine serum (FBS). The His-tag labeled RBD proteins of SARS-CoV-2 and Omicron variants were purchased from Sino Biological. Genes of bispecific antibodies were synthesized by GeneScript. The authentic Omicron with R346K mutation (BA.1.1) used in this study were isolated from COVID-19 patients in Guangzhou, passaged, and titered on Vero E6 cells. African green monkey kidney-derived Vero E6 cells were grown in DMEM supplemented with 10% FBS. Ethical approval for this study (YJ-2020-S021-01) was obtained from the Ethics Committee of the Shanghai Public Health Clinical Center. All participants in this study signed a written informed consent documents approved by the Investigational Review Board (IRB). All work with authentic SARS-CoV-2 was conducted at the Guangzhou Customs Technology Center Biosafety Level 3 (BSL-3) Laboratory.

### Production of pseudoviruses

Spike genes of SARS-CoV-2 (NC_045512), Alpha (containing 69–70 and 144 deletions and N501Y, A570D, D614G, P681H, T716I, S982A, and D1118H substitutions), Beta (containing D80A, D215G, 241–243 deletions and K417N, E484K, N501Y, D614G, and A701V substitutions), Gamma (containing L18F, T20N, P26S, D138Y, R190S, K417T, E484K, N501Y, D614G, H655Y, T1027I, and V1176F substitutions), Delta (containing T19R, 157–158 deletions and L452R, T478K, D614G, P681R, and D950N substitutions), and Omicron BA.1 (containing A67V, 69–70del, T95I, G142D, 143–145 deletions, 211 deletion, L212I, ins215EPE, G339D, S371L, S373P, S375F, K417N, N440K, G446S, S477N, T478K, E484A, Q493R, G496S, Q498R, N501Y, Y505H, T547K, D614G, H655Y, N679K, P681H, N764K, D796Y, N856K, Q954H, N969K, and L981F substitutions), BA.2 (containing T19I, A27S, 24–26 deletions, G142D, V213G, G339D, S371F, S373P, S375F, T376A, D405N, R408S, K417N, N440K, S477N, T478K, E484A, Q493R, Q498R, N501Y, Y505H, D614G, H655Y, N679K, P681H, N764K, D796Y, Q954H, and N969K), SARS-CoV, bat SARSr-CoVs (WIV1 and Rs3367) were synthesized and codon optimized by BGI and were cloned into pcDNA3.1 vector. Pseudoviruses were generated by co-transfection of 293T cells with an env-deficient HIV backbone pNL4-3.Luc.RE backbone and a spike expressing vector^22,35^. Single point mutations of Omicron were introduced into spike genes by site-directed mutagenesis as previously described^27^.

### Neutralization assay

The neutralization activities of mAbs and bispecific antibodies were determined using a single-round pseudovirus infection of Huh-7 cells. As previously described^27^, 10 μL of 5-fold serially diluted antibody was mixed and incubated with 40 μL of pseudovirus in 96-well plate at 37 °C for 1 h. 10^4^ Huh-7 cells were then added to the mixture and cultured for 48 h at 37 °C. Cells were lysed and firefly luciferase activity was detected with a luciferase assay system (Promega) on a luminometer (Perkin Elmer). The IC_50_ values of NAbs were calculated using the GraphPad Prism 7.04 software (La Jolla, CA, USA).

### Memory B cell staining, sorting, and antibody cloning

Peripheral blood mononuclear cells (PBMCs) were isolated from whole blood of recovered COVID-19 patients using density gradient centrifugation. Memory B cells (CD19^+^IgA^−^IgD^−^IgM^−^) were sorted out from PBMCs and expanded in vitro in IMDM medium supplemented with 10% FBS, IL-2, IL-21, and in the presence of irradiated 3T3-msCD40L feeder cells as previously described^36^. After 15 days of incubation, supernatants were screened for neutralization against SARS-CoV-2. From the wells with SARS-CoV-2 neutralization activities, the variable regions of the antibody (VH and VL) genes were amplified by RT-PCR. mAbs were expressed as human IgG1 by HEK293F cells and purified using a protein G column (Smart-Lifesciences).

### ELISA

2 μg/mL of SARS-CoV-2 RBD-His, Omicron trimer-His, and Omicron RBD-His protein was coated overnight at 4 °C in a 96-well plate (MaxiSorp Nunc-immuno, Thermo Scientific, USA). Wells were blocked with 5% non-fat milk (Biofroxx, Germany) and incubated with 5-fold serially diluted mAb in disruption buffer (PBS, 5% FBS, 2% BSA, and 1% Tween-20) for 1 h subsequently. After 3 washing steps with PBST (PBS, 0.05% Tween-20), the binding antibodies were detected by 1:2500 diluted HRP-conjugated goat anti-human IgG antibody (Jackson Immuno Research Laboratories, USA). Plates were washed three times with 0.2% Tween-20 in PBS and developed using ABST (Thermo Scientific, USA) for 30 min. Absorbance at 405 nm was read on a Multiskan FC plate reader (Thermo Scientific, USA).

### BLI binding assay and competition assay

BLI experiments were carried out on a FortéBio OctetRED96 instrument. The kinetics of mAb binding to SARS-CoV-2 RBD-His, Omicron RBD-His, or trimer-His proteins was measured using anti-human IgG (AHC) biosensors. 10 μg/mL of mAbs were immobilized on biosensors for 200 s. After a 120-s stabilization step with 0.02% PBST (PBS with 0.02% Tween), biosensors were moved into 6 μg/mL of SARS-CoV-2 RBD-His, Omicron RBD-His, or trimer-His proteins for the 300-s association step. Then biosensors were moved into 0.02% PBST to detect dissociation for 300 s. The *K*_on_, *K*_off_, and *K*d of antibody with RBD proteins were calculated by FortéBio Data Analysis software (Version 8.1) using 1:1 binding and a global fitting model.

For the antibody competition assay, 10 μg/mL of mAb1 was immobilized on the anti-human IgG (AHC) biosensors for 200 s. After wash with 0.02% PBST for 120 s to reach baseline, biosensors were moved into 50 μg/mL of IgG1 isotype control for 200 s and then moved into SARS-CoV-2 RBD at 6 μg/mL for 300 s. After wash with 0.02% PBST for 120 s, biosensors were moved into 10 μg/mL of mAb2 for 600 s to detect the association between mAb2 and SARS-CoV-2 RBD.

The antibody inhibition of RBD binding to ACE2 was measured as previously described^27^. Briefly, biosensors were loaded with ACE2-Fc for 600 s and moved into pre-mix of mAb and SARS-CoV-2 RBD at a 6:1 molar ratio for 600 s after blocking. We set the mixture of ACE2-Fc and SARS-CoV-2 RBD as a positive control, and IgG1 isotype control.

### Focus reduction neutralization test

SARS-CoV-2 Omicron BA.1.1 focus reduction neutralization test was performed in a certified biosafety level 3 lab. 15 μL of serially diluted antibodies were mixed with 50 μL of SARS-CoV-2 (100 focus forming unit) and incubated at 37 °C for 1 h. Mixtures were then transferred to 96-well plates seeded with Vero E6 cells (ATCC, Manassas, VA) for 1 h at 37 °C to allow virus entry. The inoculums were replaced by overlay media (100 μL MEM containing 1.2% carboxymethylcellulose) and incubated for additional 24 h. Then the overlay was discarded and the cell monolayer was fixed with 4% paraformaldehyde solution for 2 h at room temperature (RT). After being permeabilized with 0.2% Triton X-100 for 20 min at RT, the plates were sequentially stained with cross-reactive rabbit anti-SARS-CoV-2 N IgG (Cat# 40143-T62, Sino Biological Inc.) as the primary antibody and HRP-conjugated goat anti-rabbit IgG(H + L) (109-035-088, Jackson ImmunoResearch) as the secondary antibody at 37 °C for 1 h, respectively. The numbers of SARS-CoV-2 foci were calculated using CTL ImmunoSpot S6 Ultra reader (Cellular Technology Ltd.) after development with KPL TrueBlue Peroxidase substrates. Neutralizing activity was defined as the ratio of inhibition of SARS-CoV-2 focus comparing diluted antibody to control.

### Construction and expression of bispecific NAbs

Bispecific antibodies consisting of the scFv of GW01 and scFv of 16L9, REGN10987 or 4L12 were constructed as previously described^27^. Briefly, the variable light chain (VL) and variable heavy chain (VH) of antibodies were linked with GlySer linkers in order and fused to the expression vector with hinge-CH2-CH3 fragment of human immunoglobulin (hIgG1 Fc). FD01 bispecific antibody sequence order was as follows: GW01 VL-(Gly_4_Ser)_3_-GW01 VH-GlySer(Gly_4_Ser)_4_-16L9 VL-(Gly_4_Ser)_3_-16L9 VH-hinge-CH2-CH3.

HEK293F cells were transiently transfected with bispecific antibody plasmid. After 6 days of culture at 37 °C in a 5% CO_2_ incubator, the supernatant was collected and filtered. Bispecific antibodies were purified with protein G column (Smart-Lifesciences) and stored in PBS at −80 °C.

### SDS-PAGE and cross-linking SDS-PAGE of bispecific antibodies

The purity of bispecific antibodies was analyzed by SDS-PAGE and Coomassie staining as previously described^27^. Briefly, 5× SDS loading sample buffer containing 10% β-mercaptoethanol was mixed with 5 μg of each bispecific antibody. After heating for 10 min at 100 °C, samples were then loaded on a 4%–20% SDS gradient gel (GeneScript Biotech Corporation). The gel was run for 120 min at 120 V, and Coomassie staining was performed.

The extent of dimer of bispecific antibodies was investigated by cross-linking of bispecific antibodies with glutaraldehyde (Sigma-Aldrich). Briefly, 0.2 μg/μL PBS-diluted bispecific antibodies were cross-linked in the presence of 2.7 µM glutaraldehyde cross-linker at RT for 5 min. The reaction was quenched by adding 1 M Tris-HCl buffer (pH 8.0) to a final concentration of 40 mM. The cross-linked antibodies were analyzed on 4%–20% SDS gradient gel and stained by Coomassie blue as previously described^27^.

### Expression and purification of SARS-CoV-2 Omicron spike

The Human codon-optimized gene encoding Omicron S ectodomain was purchased from GeneScript. The Omicron S 6P substitution^37^ was constructed into expression vector pcDNA3.1 and transfected into HEK293F suspension using polyethlenimine. The supernatants were harvested after 72 h, and then purified by affinity column Histrap HP (GE Healthcare). The protein was then further purified using gel filtration column Superose 6 increase 10/300 GL (GE Healthcare) in buffer containing 20 mM Tris (pH 8.0) and 200 mM NaCl.

### Cryo-EM sample preparation

Purified Omicron S protein at 0.55 mg/mL was mixed with IgG FD01 with a molar ratio of 1:1.5, and then incubated on ice for 10 min before being added to freshly plasma-cleaned gold grids^38^ (holey amorphous nickel-titanium alloy film, 400 mesh, R1.2/1.3). The sample was then quickly frozen by using Vitrobot IV (Thermo Fisher Scientific), with 2 s blot time and −3 blot force and 10 s wait time.

### Cryo-EM data collection and image processing

Cryo-EM data of Omicron S with FD01 antibody were captured on a Titan Krios TEM (Thermo Fisher Scientific) equipped with a K3 camera (Gatan) and an energy filter (GIF quantum, Gatan). Movie stacks were collected automatically with SerialEM package^39^ through beam-image shift method^40^ at the super-resolution mode with a physical pixel size of 1.064 Å. Defocus ranges were set from −1.2 μm to −2.5 μm, and each movie stack was exposed for 3 s with a total dose of ~58 e^−^/Å^2^.

Totally 4363 movie stacks were imported into RELION3.0^41^, then motion corrected (binned by 2 and dose-weighted) by MotionCor2^42^, and contrast transfer function (CTF) was estimated by Gctf^43^. After manual selection, 3817 good images were imported into cryoSPARC^9^ for further blob-picking and 2D classification. Good 2D classes were further used as templates for template-picking. During 2D classification, trimer dimer and trimer were observed, good particles were merged and deduplicated from blob-picking and template-picking and subsequently exported back to RELION through pyem package^44^.

For trimer dimer particles, 1,003,956 particles were extracted at a larger box-size of 540 and rescaled to 180. After performing 2 rounds of 3D classification with a soft circular mask of 480 Å in diameter in RELION, 166,441 particles were selected and re-extracted unbinned (1.064 Å/pixel), and then auto-refined without applying symmetry, yielding a map at the resolution of 6.11 Å.

For trimer map, 1,003,956 particles were subjected to another extraction at a box-size of 320 and rescaled to 160. These particles were then subjected to 1 round of 3D classification with a soft circular mask of 220 Å. Three classes with different conformations were selected separately for another round of 3D classification. Particles in different states were auto-refined, CTF-refined, and polished separately. The density of RBDs and FD01 in some states were not well-resolved, and therefore we carried out no-alignment 3D classification with NTD-RBD-Fabs (NRF) mask to improve those regions. Finally, we got 5 states of Omicron S–FD01 trimer.

To get more details about the interface of RBD and FD01, local-refinement was performed focusing on this region. Good 3D-classes were selected with relatively complete RBD and FD01 density within all states. We auto-refined these particles with a C3-aligned reference, but the auto-refinement procedure was not applied with any symmetry. Then particles were expanded with C3 symmetry and further subtracted with one NRF mask. After no-alignment 3D-classification, 249,122 particles with complete NRF density were selected out, exported to cryoSPARC and subjected to local refinement, yielding a local-refined map at 3.51 Å.

The resolution of all maps was estimated based on the gold-standard Fourier shell correlation (FSC) 0.143 criterion. The above procedures of data processing are summarized in Supplementary Figs. S3, S4. These sharpened maps were generated by DeepEMhancer^45^ and then handedness was corrected in UCSF Chimera^13^ for subsequent model building and analysis.

### Model building and refinement

For model building of these maps, Omicron S trimer model and the antibody model were generated by swiss-model^46^, then fitted into the map using UCSF Chimera and subsequently manually adjusted with COOT^47^. After that, real-space refinement was further carried out in PHENIX^48^. The local refinement map was used to get more precise model of RBD bound with 16L9 and GW01, and then docked back into global refinement trimer and trimer dimer maps. Model validation was performed using MolProbity. Figures were prepared using UCSF Chimera and UCSF ChimeraX^49^.

## Supplementary information

Supplementary information

Supplementary Video S1

## Data Availability

The cryo-EM maps and the coordinates of SARS-CoV-2 Omicron S complexed with FD01 have been deposited to the Electron Microscopy Data Bank (EMDB) and Protein Data Bank (PDB) with accession numbers EMD-32655 and PDB 7WOQ (state 1), EMD-32656 and PDB 7WOR (state 2), EMD-32657 and PDB 7WOS (state 3), EMD-32659 and PDB 7WOU (state 4), EMD-32660 and PDB 7WOV (state 5), EMD-32661 and PDB 7WOW (state 6), EMD-32654 and PDB 7WOP (NTD-RBD-GW01-16L9 local refinement). The accession numbers for the nucleotide and protein sequences for variable regions of GW01, 4L12 and 16L9 in GenBank are OP480801–OP480806.

## References

[1] WHO. https://www.who.int/news/item/26-11-2021-classification-of-omicron-(b.1.1.529)-sars-cov-2-variant-of-concern (2021).

[2] Yamasoba D (2022). Virological characteristics of the SARS-CoV-2 Omicron BA.2 spike. Cell.

[3] Cameroni E (2022). Broadly neutralizing antibodies overcome SARS-CoV-2 Omicron antigenic shift. Nature.

[4] Cele S (2022). Omicron extensively but incompletely escapes Pfizer BNT162b2 neutralization. Nature.

[5] Liu L (2022). Striking antibody evasion manifested by the Omicron variant of SARS-CoV-2. Nature.

[6] Planas D (2022). Considerable escape of SARS-CoV-2 Omicron to antibody neutralization. Nature.

[7] Rössler A, Riepler L, Bante D, von Laer D, Kimpel J (2022). SARS-CoV-2 Omicron variant neutralization in serum from vaccinated and convalescent persons. N. Engl. J. Med..

[8] Carreño JM (2022). Activity of convalescent and vaccine serum against SARS-CoV-2 Omicron. Nature.

[9] Garcia-Beltran WF (2022). mRNA-based COVID-19 vaccine boosters induce neutralizing immunity against SARS-CoV-2 Omicron variant. Cell.

[10] Dejnirattisai W (2022). Reduced neutralisation of SARS-CoV-2 omicron B.1.1.529 variant by post-immunisation serum. Lancet.

[11] Wang Y (2022). Resistance of SARS-CoV-2 Omicron variant to convalescent and CoronaVac vaccine plasma. Emerg. Microbes Infect..

[12] Dejnirattisai W (2021). The antigenic anatomy of SARS-CoV-2 receptor binding domain. Cell.

[13] Cao Y (2020). Potent neutralizing antibodies against SARS-CoV-2 identified by high-throughput single-cell sequencing of convalescent patients’ B cells. Cell.

[14] Rappazzo CG (2021). Broad and potent activity against SARS-like viruses by an engineered human monoclonal antibody. Science.

[15] Li D (2021). In vitro and in vivo functions of SARS-CoV-2 infection-enhancing and neutralizing antibodies. Cell.

[16] Tortorici MA (2021). Broad sarbecovirus neutralization by a human monoclonal antibody. Nature.

[17] Liu L (2020). Potent neutralizing antibodies against multiple epitopes on SARS-CoV-2 spike. Nature.

[18] Cerutti G (2021). Neutralizing antibody 5-7 defines a distinct site of vulnerability in SARS-CoV-2 spike N-terminal domain. Cell Rep..

[19] Hansen J (2020). Studies in humanized mice and convalescent humans yield a SARS-CoV-2 antibody cocktail. Science.

[20] Jones BE (2021). The neutralizing antibody, LY-CoV555, protects against SARS-CoV-2 infection in nonhuman primates. Sci. Transl. Med..

[21] Shi R (2020). A human neutralizing antibody targets the receptor-binding site of SARS-CoV-2. Nature.

[22] Ju B (2020). Human neutralizing antibodies elicited by SARS-CoV-2 infection. Nature.

[23] Banach BB (2021). Paired heavy- and light-chain signatures contribute to potent SARS-CoV-2 neutralization in public antibody responses. Cell Rep..

[24] Kim C (2021). A therapeutic neutralizing antibody targeting receptor binding domain of SARS-CoV-2 spike protein. Nat. Commun..

[25] Zost SJ (2020). Potently neutralizing and protective human antibodies against SARS-CoV-2. Nature.

[26] Dejnirattisai W (2022). SARS-CoV-2 Omicron-B.1.1.529 leads to widespread escape from neutralizing antibody responses. Cell.

[27] Wang Y (2022). Novel sarbecovirus bispecific neutralizing antibodies with exceptional breadth and potency against currently circulating SARS-CoV-2 variants and sarbecoviruses. Cell Discov..

[28] Zhou T (2022). Structural basis for potent antibody neutralization of SARS-CoV-2 variants including B.1.1.529. Science.

[29] VanBlargan LA (2022). An infectious SARS-CoV-2 B.1.1.529 Omicron virus escapes neutralization by therapeutic monoclonal antibodies. Nat. Med..

[30] Iketani S (2022). Antibody evasion properties of SARS-CoV-2 Omicron sublineages. Nature.

[31] Mannar D (2021). Structural analysis of receptor binding domain mutations in SARS-CoV-2 variants of concern that modulate ACE2 and antibody binding. Cell Rep..

[32] Wu X (2018). Tandem bispecific neutralizing antibody eliminates HIV-1 infection in humanized mice. J. Clin. Invest..

[33] Wang Y (2022). A broadly neutralizing antibody against SARS-CoV-2 Omicron variant infection exhibiting a novel trimer dimer conformation in spike protein binding. Cell Res..

[34] De Gasparo R (2021). Bispecific IgG neutralizes SARS-CoV-2 variants and prevents escape in mice. Nature.

[35] Wu F (2020). Evaluating the association of clinical characteristics with neutralizing antibody levels in patients who have recovered from mild COVID-19 in Shanghai, China. JAMA Intern. Med..

[36] Huang J (2013). Isolation of human monoclonal antibodies from peripheral blood B cells. Nat. Protoc..

[37] Hsieh CL (2020). Structure-based design of prefusion-stabilized SARS-CoV-2 spikes. Science.

[38] Huang X (2020). Amorphous nickel titanium alloy film: a new choice for cryo electron microscopy sample preparation. Prog. Biophys. Mol. Biol..

[39] Mastronarde DN (2005). Automated electron microscope tomography using robust prediction of specimen movements. J. Struct. Biol..

[40] Wu C, Huang X, Cheng J, Zhu D, Zhang X (2019). High-quality, high-throughput cryo-electron microscopy data collection via beam tilt and astigmatism-free beam-image shift. J. Struct. Biol..

[41] Zivanov J (2018). New tools for automated high-resolution cryo-EM structure determination in RELION-3. Elife.

[42] Zheng SQ (2017). MotionCor2: anisotropic correction of beam-induced motion for improved cryo-electron microscopy. Nat. Methods.

[43] Zhang K (2016). Gctf: Real-time CTF determination and correction. J. Struct. Biol..

[44] Asarnow, D., Palovcak, E. & Cheng, Y. UCSF pyem v0.5. Zenodo 10.5281/zenodo.3576630 (2019).

[45] Sanchez-Garcia R (2021). DeepEMhancer: a deep learning solution for cryo-EM volume post-processing. Commun. Biol..

[46] Waterhouse A (2018). SWISS-MODEL: homology modelling of protein structures and complexes. Nucleic Acids Res..

[47] Emsley P, Lohkamp B, Scott WG, Cowtan K (2010). Features and development of Coot. Acta Crystallogr. D Biol.Crystallogr..

[48] Afonine PV (2018). Real-space refinement in PHENIX for cryo-EM and crystallography. Acta Crystallogr. D Struct. Biol..

[49] Pettersen EF (2021). UCSF ChimeraX: structure visualization for researchers, educators, and developers. Protein Sci..

