# Supplementary material for: Combating the SARS-CoV-2 Omicron (BA.1) and BA.2 with potent bispecific antibodies engineered from non-Omicron neutralizing antibodies
Source: Cell Discov. 2022 Oct 7;8:104. doi: 10.1038/s41421-022-00463-6 (PMC9540141; doi:10.1038/s41421-022-00463-6)
Supplement: Supplementary file 1 — Supplementary information [file 41421_2022_463_MOESM1_ESM.pdf]

Supplementary Materials for

Combating the SARS-CoV-2 Omicron (BA.1) and BA.2 with potent bispecific antibodies engineered from non-Omicron neutralizing antibodies

Yingdan Wang[1#], Xiang Zhang[1#], Yunping Ma[1,2#], Yanqun Wang[3#], Wuqiang Zhan[1], Qinwen Zheng[1], Meng Zhang[1], Ping Ji[1], Mei Liu[1], Qianying Liu[1], Tingting Sun[1], Tongyu Zhu[1], Yumei Wen[1], Lei Sun[1]*, Jincun Zhao[3,4,5]*, Fan Wu[2]*, Zhenguo Chen[1]*, Jinghe Huang[1]*

[1]Key Laboratory of Medical Molecular Virology (MOE/NHC/CAMS) and Shanghai Institute of Infectious Disease and Biosecurity, the Fifth People's Hospital of Shanghai, Shanghai Public Health Clinical Center, Institutes of Biomedical Sciences, School of Basic Medical Sciences, Fudan University, Shanghai, China.

[2]Shanghai Immune Therapy Institute, Shanghai Jiao Tong University School of Medicine Affiliated Renji Hospital, Shanghai, China.

[3]State Key Laboratory of Respiratory Disease, National Clinical Research Center for Respiratory Disease, Guangzhou Institute of Respiratory Health, the First Affiliated Hospital of Guangzhou Medical University, Guangzhou, Guangdong, China;

[4]Institute of Infectious Disease, Guangzhou Eighth People's Hospital of Guangzhou Medical University, Guangzhou, Guangdong, China.

[5]Guangzhou Laboratory, Bio-island, Guangzhou 510320, China

[#]These authors contributed equally.

*Correspondence to: llsun@fudan.edu.cn, zhaojincun@gird.cn, wufan@renji.com, ZhenguoChen@fudan.edu.cn, or jinghehuang@fudan.edu.cn.

Keywords: COVID-19; SARS-CoV-2; Bispecific antibodies; Omicron; BA.2

Table S1. Mutations in spike proteins of Omicron, BA.1.1, BA.2 and BA.3.

Spkie		Muation	BA.1	BA.1.1	BA.2	BA.3
NTD		T19I			■	
		Δ24-26			■	
		A27S			■	
		A67V	■	■		■
		Δ69-70	■	■		■
		T95I	■	■		■
		G142D	■	■	■	■
		Δ143-145	■	■		■
		Δ211	■	■		■
		L212I	■	■		■
		V213G			■	
		ins214EPE	■	■		
RBD		G339D	■	■	■	■
		R346K		■		
		S371L/F	L	L	F	F
		S373P	■	■	■	■
		S375F	■	■	■	■
		T376A			■	
		D405N			■	■
		R408S			■	
		K417N	■	■	■	■
	RBM	N440K	■	■	■	■
		G446S	■	■		■
		S477N	■	■	■	■
		T478K	■	■	■	■
		E484A	■	■	■	■
		Q493R	■	■	■	■
		G496S	■	■		
		Q498R	■	■	■	■
		N501Y	■	■	■	■
		Y505H	■	■	■	■
SD1		T547K	■	■		
SD2		D614G	■	■	■	■
		H655Y	■	■	■	■
		N679K	■	■	■	■
		P681H	■	■	■	■
FP		N764K	■	■	■	■
		D796Y	■	■	■	■
		N856K	■	■		
HR1		Q954H	■	■	■	■
		N969K	■	■	■	■
		L981F	■	■		

Table S2. The germline and CDRH3 sequences of GW01, 4L12, and 16L9.

Donor ID	mAb ID	VH	CDRH3 sequence	VL	CDRL3 sequence
Donor 1	GW01	IGHV3-43	AKDRSYGPPDVFNYEYGMDV	IGLV1-44	AAWDDSLNWV
Donor 1	4L12	IGHV3-66	ARDLITYGMDV	IGKV1-9	QQLNSYPPLT
Donor 2	16L9	IGHV3-53	ARGEIQPYYYYGMDV	IGLV2-8	SSYAGSSNFDV

Table S3. Cryo-EM data collection and refinement statistics of SARS-CoV-2 complexed with IgG FD01.

	State 1	State 2	State 3	State 4	State 5	State 6	Local refine NRF
**Data collection and processing**							
Magnification				81,000			
Voltage (kV)				300			
Electron exposure (e–/Å^2)				58			
Defocus range (μm)				-1.2 to -2.5			
Pixel size (Å)				1.064			
Initial particles (no.)				1,003,956			
Symmetry imposed				C1			
Final particles (no.)	194,026	62,040	74,415	141,577	39,294	71,568	249,122
Map resolution (Å)	3.47	3.70	3.91	3.47	3.87	6.11	3.51
**Refinement**							
R.m.s. deviations							
Bond lengths (Å)	0.003	0.003	0.003	0.003	0.003	0.002	0.002
Bond angles (°)	0.539	0.506	0.499	0.557	0.527	0.437	0.524
Validation							
MolProbity score	2.52	2.48	2.46	2.50	2.53	2.41	2.88
Clashscore	9.18	8.06	8.38	8.44	8.14	7.67	10.97
Rotamer outlier (%)	5.14	5.34	5.30	5.39	5.97	5.33	9.21
Ramachandran plot							
Favored (%)	91.75	92.00	92.74	91.84	91.67	93.20	88.62
Allowed (%)	7.95	7.90	7.14	8.13	8.17	6.80	11.38
Disallowed (%)	0.30	0.11	0.13	0.03	0.16	0.00	0.00
**EMDB**	32655	32656	32657	32659	32660	32661	32654
**PDB**	7WOQ	7WOR	7WOS	7WOU	7WOV	7WOW	7WOP

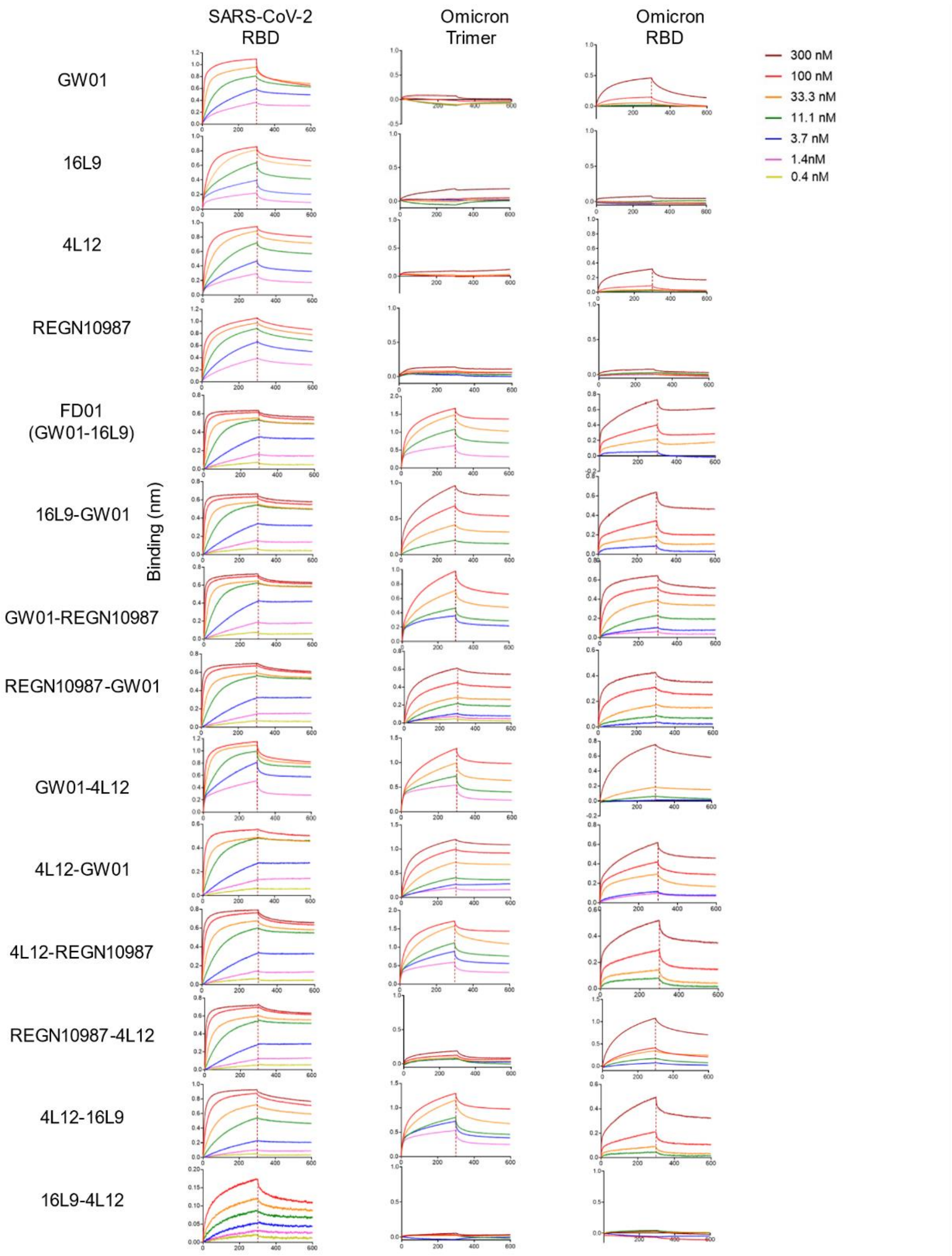

Fig. S1. Binding affinities of GW01, 16L9, 4L12, REGN10987, and ten bispecific antibodies to SARS-CoV-2 RBD-his, Omicron trimer-his and Omicron RBD-his measured by bilayer interferometry. Antibodies were immobilized on anti-human IgG (AHC) biosensors and then tested for their binding abilities to the target proteins.

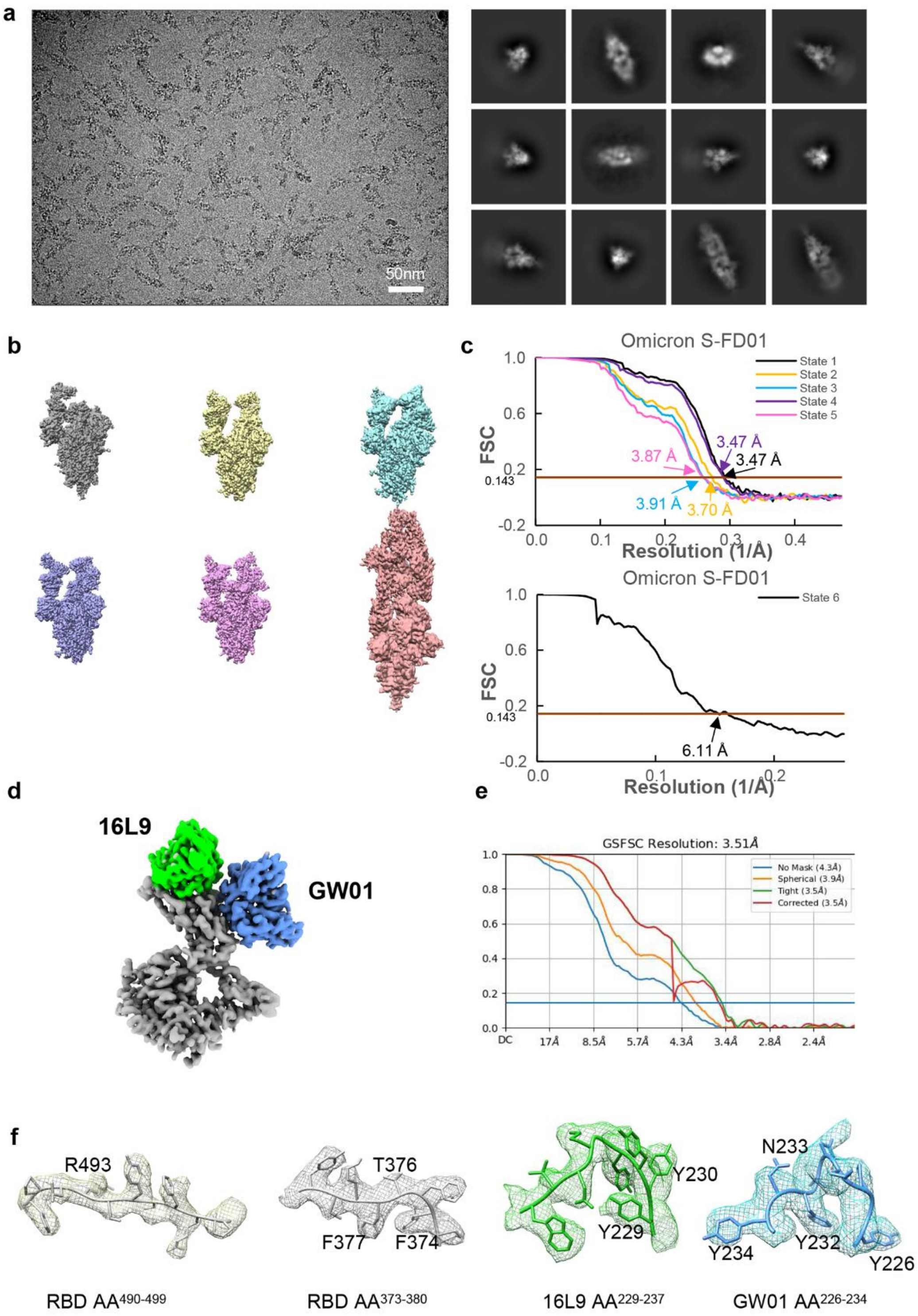

Fig. S2. Cryo-EM data collection and processing of FD01 bound SARS-CoV-2 Omicron S. (a) Representative electron micrograph and 2D classification results of FD01 bound SARS-CoV-2 S. **(b)** The reconstruction map of the complex structures at

six states. **(c)** Gold-standard Fourier shell correlation curves generated in RELION for structures of six states. The 0.143 cut-off is indicated by a horizontal dashed line. **(d)** The local-refined map of the NRF region. **(e)** Gold-standard Fourier shell correlation curves generated in cryoSPARC for local-refined map. (**f**) Density maps of residues around the interface.

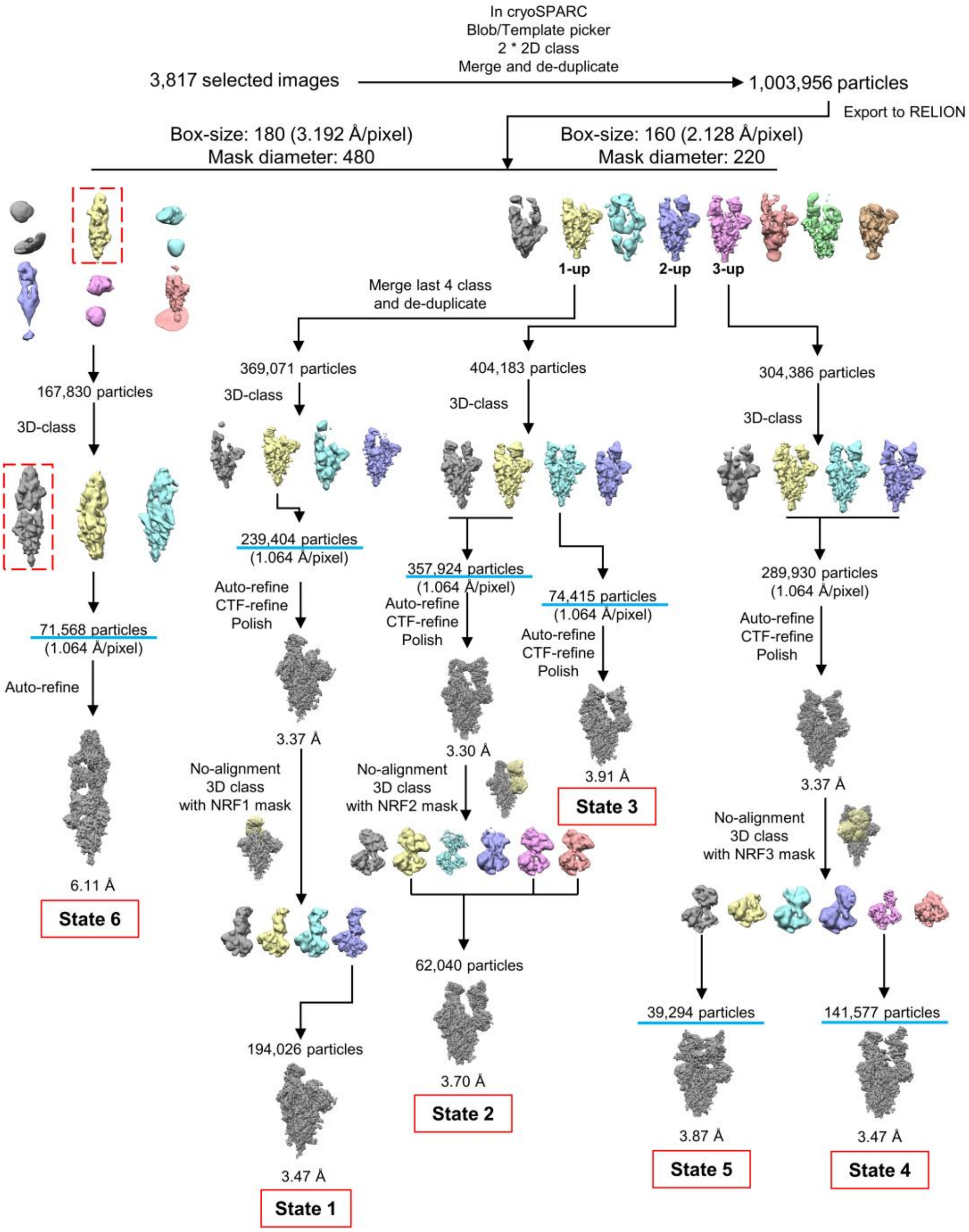

Fig. S3. Data processing flowchart of FD01 bound SARS-CoV-2 Omicron S trimer. Particles number above cyan line is used for particle counting statistics.

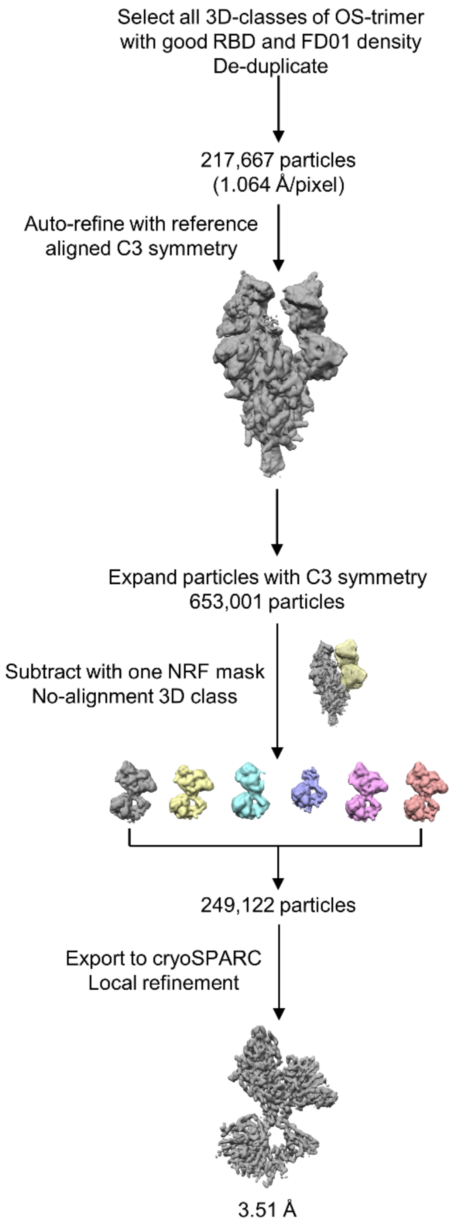

Fig. S4. Data processing flowchart of local refinement of RBD-FD01.

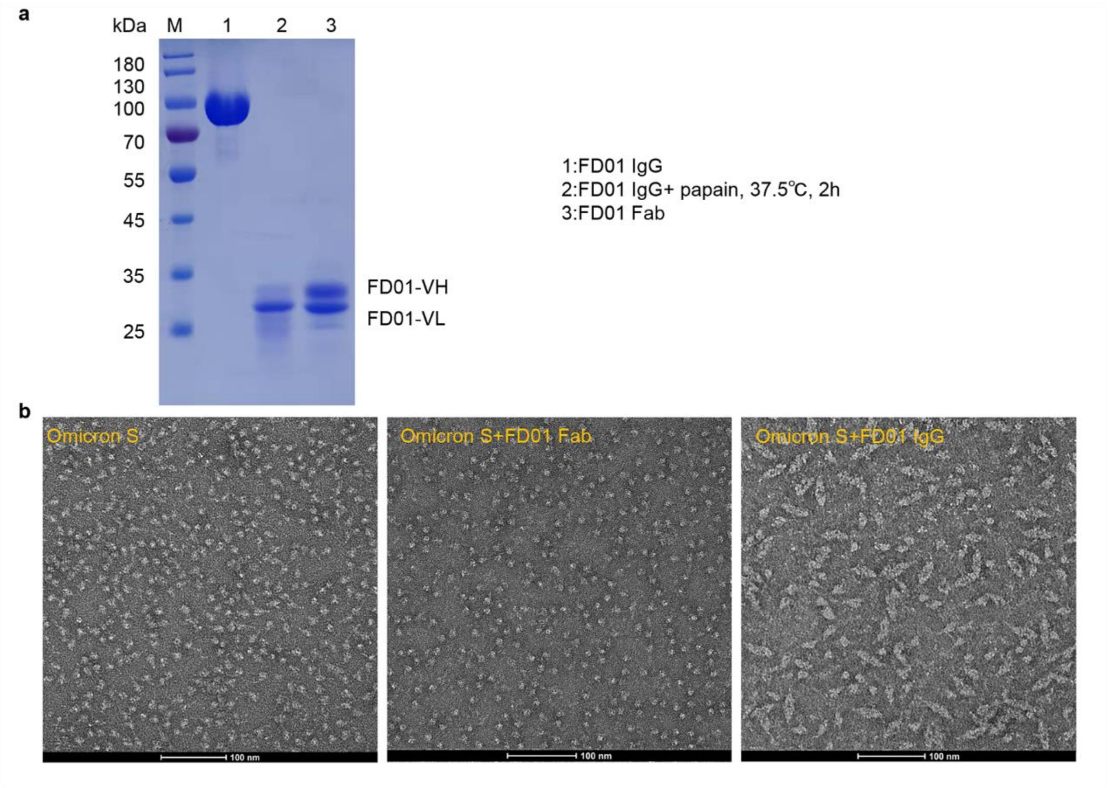

Fig. S5. IgG FD01 crosslinks Omicron S trimers to form trimer dimer conformation. (**a**) SDS-PAGE of the FD01 in IgG and Fab form; (**b**) Negative stain images of Omicron S trimer-FD01 Fab and Omicron S trimer-FD01 IgG, showing that only IgG FD01 can induce the formation of trimer dimer.

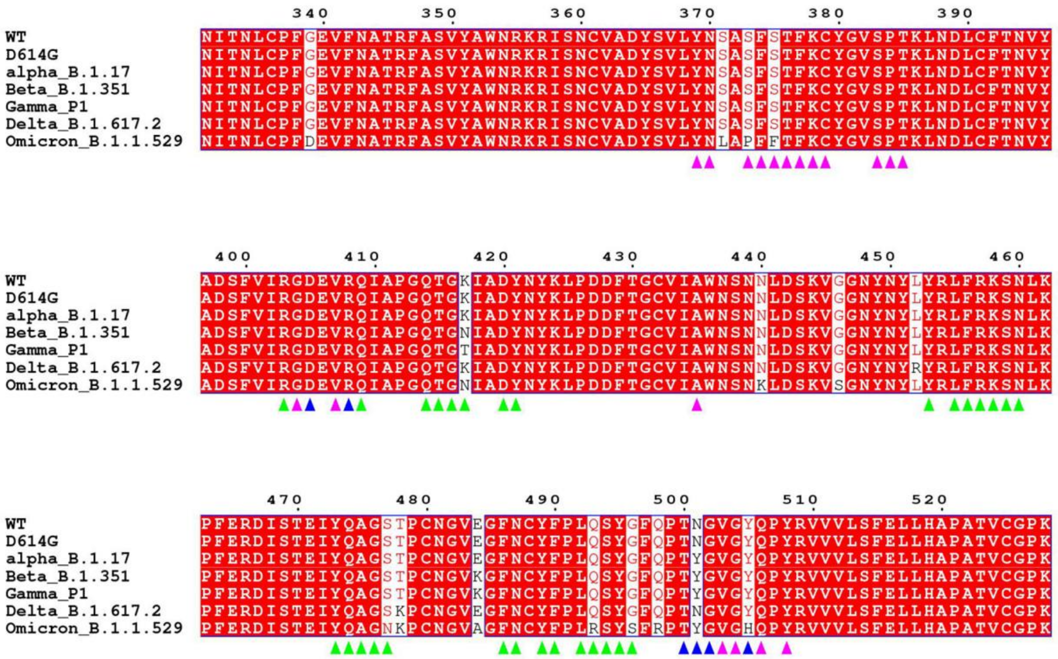

Fig. S6. Sequence alignment of SARS-CoV-2 WT and all VOCs RBDs. Conserved amino acids are highlighted as red. Residues involved in 16L9 or GW01 are marked with triangles in green or magenta, respectively. Residues involved in both 16L9 and GW01 binding are marked with triangles in blue.

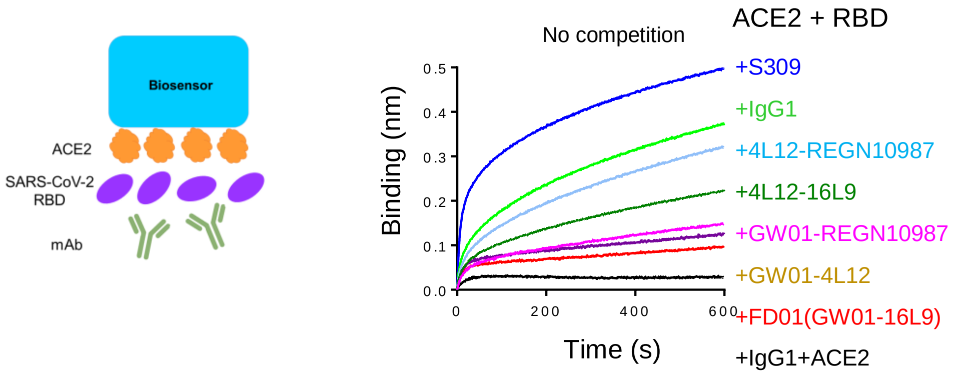

Fig. S7. Five representative bispecific antibodies block RBD binding to ACE2.
Binding of ACE2 to the SARS-CoV-2 RBD in competition with bispecific antibodies (red), S309 (blue), control IgG1 (green), and IgG1+ACE2 (black).

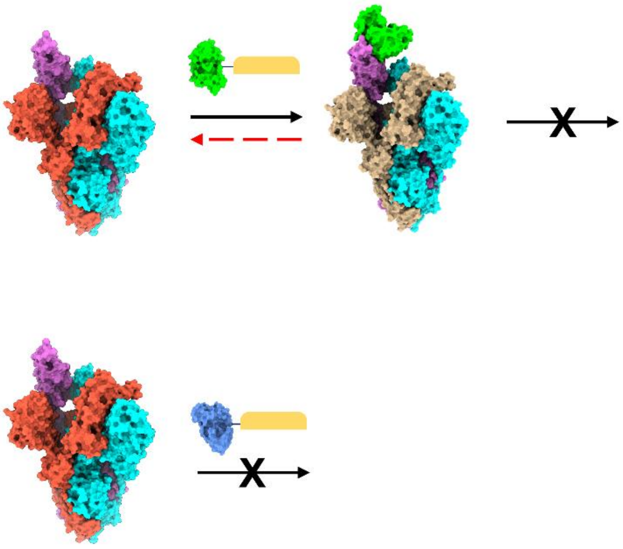

Fig. S8. Hypothesis of binding features when Omicron S trimer meets with mAbs of 16L9 or GW01.

Supplementary Video: Conformation transitions of Omicron S trimer and the representative bispecific antibody GW01-16L9 (FD01) in six states.
